# Supplementary material for: Influence of Magnesium Ions on the Preparation and Storage of DNA Tetrahedrons in Micromolar Ranges
Source: Molecules. 2019 Jun 1;24(11):2091. doi: 10.3390/molecules24112091 (PMC6600315; doi:10.3390/molecules24112091)
Supplement: Supplementary file 1 [file molecules-24-02091-s001.pdf]

Magnesium ion influence on the preparation and storage of DNA tetrahedrons in micromolar ranges

Yue Hu[1, †], Zhou Chen[1, †], Zheng Hou[1], Mingkai Li[1], Bo Ma[1], Xiaoxing Luo[1,*], Xiaoyan Xue[1,*]

Department of pharmacology, Fourth Military Medical University, Xi'an, China; yuehu918@163.com (Y.H.); chenzhou_cky@163.com (Z.C.); hzh_0001@163.com (Z.H.); mingkai@fmmu.edu.cn (M.L.); mbcarl@163.com (B.M.); xxluo3@fmmu.edu.cn (X.L.); xxy.0707@163.com (X.X)

* Correspondence: xxluo3@fmmu.edu.cn; xxy.0707@163.com; Tel: 18992845576(X.L.); 13891968541(X.X)

† Both authors contributed equally to this work.

Table S1. The sequences of DNA tetrahedrons.

DNA	Sequence
S1	AGGCAGTTGAGACGAACATTCCTAAGTCTGAAATTTATCACCCGCCATAGTAGAC GTATCACC
S2	CTTGCTACACGATTCAGACTTAGGAATGTTCGACATGCGAGGGTCCAATACCGAC GATTACAG
S3	GGTGATAAAACGTGTAGCAAGCTGTAATCGACGGGAAGAGCATGCCCATCCACT ACTATGGCG
S4	CCTCGCATGACTCAACTGCCTGGTGATACGAGGATGGGCATGCTCTTCCCGACGG TATTGGAC

Table S2. The polydispersity index (PDI) of DNA particles prepared in different conditions determined by DLS. (Data were mean±SD, n = 3).

Td concentrations	Mg^{2+} concentrations (mM)				
(μM)	2	5	10	25	50
1	0.29±0.05	0.28±0.06	0.39±0.06	0.26±0.01	0.40±0.10
2	0.37±0.11	0.29±0.05	0.33±0.04	0.26±0.11	0.43±0.15
5	0.32±0.07	0.29±0.03	0.37±0.09	0.35±0.18	0.61±0.34
10	0.39±0.10	0.35±0.14	0.35±0.15	0.37±0.24	0.48±0.27
20	0.37±0.02	0.37±0.17	0.55±0.20	0.46±0.21	0.88±0.39

Table S3. Yields of DNA tetrahedrons under different conditions (%). (Data were mean±SD, n = 3).

Td concentrations	Mg^{2+} concentrations (mM)						
(μM)	0.05	0.5	2	5	10	25	50
1	17±1	55±4	77±5	80±4	73±2	52±1	50±2
2	15±0	43±2	71±2	80±3	67±3	49±2	49±3
5	14±0	44±5	75±3	70±1	69±2	53±2	26±2
10	12±2	41±1	72±0	70±2	63±2	22±2	19±2
20	10±0	18±0	55±2	45±2	54±1	7±1	5±1
